# Stability Analysis of Switched Linear Singular Systems with Unstable and Stable Modes

**DOI:** 10.3390/e25091300

**Published:** 2023-09-05

**Authors:** Jiandong Xiong, Yidian Wang, Yanfang Huo, Hongpeng Zhao

**Affiliations:** College of Mathematics and Information Science, Henan Normal University, Xinxiang 453007, China

**Keywords:** switched systems, stability analysis, singular systems, time-varying piecewise Lyapunov function

## Abstract

In this paper, stability is studied for a class of switched singular systems containing both stable and unstable modes. By introducing a time-varying piecewise Lyapunov function (TVPLF) and a mode-dependent average dwell time (ADT) switching rule, the computable sufficient conditions for system stability are derived. The time-varying piecewise Lyapunov functions are piecewise continuously differentiable on every mode (but may not be differentiable at the interpolating points of the dwell time). This Lyapunov function method is particularly advantageous in overcoming the limitations of traditional multiple Lyapunov function (MLF) methods, which may not have a feasible solution when dealing with switched systems containing only unstable modes. As such, the TVPLF offers greater flexibility in application. Compared with the conventional ADT switching rule, the mode-dependent ADT switching rule not only enables each mode to have its own ADT but also allows for its own switching strategy. Specifically, the stable mode adopts a slow switching strategy while the unstable mode adopts a fast one, thereby reducing the conservatism of the ADT switching rule. Furthermore, based on the stability analysis, the time-varying controllers are proposed to stabilize the switched singular system, which can be expressed as the sequential linear combination of a series of linear state feedback on each mode. The proposed controllers are continuous for each mode, which are different from the controllers designed through the traditional MLF and MDLF methods, where the controllers designed by traditional MLF are the time-invariant linear state feedback in each mode while the controllers designed by the MDLF are piecewise continuous for each mode.

## 1. Introduction

Switched systems are usually composed of the subsystems and the switching rules that regulate the operation of each mode. As a versatile modeling tool, switched systems are widely used in industrial electronics, traffic congestion, network control, aircraft control systems, and other fields. Therefore, research on switched systems has important theoretical and practical significance. In recent decades, scholars have devoted themselves to the study of switched systems and have made many achievements [1,2,3,4,5,6,7,8,9,10,11]. Stability analysis is one of the main research topics of switched systems. A common approach to determine the stability of a switched system is by using the common Lyapunov function (CLF) [1,2,3]. In [1], some necessary and sufficient conditions were given to ensure the existence of a common quadratic Lyapunov function for switched linear systems with special structures. Then, some algebraic criteria were proposed to ensure the existence of a common quadratic Lyapunov function for switched systems in [2,3]. When the CLF method is used to analyze the stability of the switched systems, the switching rules are ignored—that is, the switched systems are stable under arbitrary switching rules if there exist common Lyapunov functions. However, switching rules play an important role in the stability analysis of switched systems. Also, it should be noted that even in cases where no CLF exists for the systems, stability can still be achieved [4,5] via some proper switching rules. Therefore, when dealing with constrained switching rules, the CLF method is proved to be too conservative. To address this limitation, the MLF approach has been proposed as an effective means to mitigate the conservatism inherent of CLF [6,7]. Based on the multiple linear copositive Lyapunov functions approach, the asymptotic stability of switched positive systems was investigated in [6,7]. However, when dealing with switched systems containing only unstable modes, it may not be feasible to find a MLF. Since all the modes are unstable, the Lyapunov function $V_{i}$ of each mode has an increment, where the subscript *i* is the label of the mode. To ensure the stability of the switched systems, the Lyapunov function is attenuated at the switching instant to suppress the increment—that is, there exists 0<μ<1 such that $V_{j}<\mu V_{i}$, supposing the mode *i* switch to mode *j* at the switching instant. When the system switches to mode *i* again, one has $V_{i}<\mu V_{k}<\ldots <\mu^{s-1}V_{j}<\mu^{s}V_{i}$, where the integer *s* is the switching time. $V_{i}<\mu^{s}V_{i}$ is a contradiction. In order to address this problem, a multiple discontinuous Lyapunov function (MDLF) approach was introduced in previous works [8]. The MDLF allows multiple Lyapunov functions for each mode instead of one Lyapunov function. During the dwell time of each mode, the discontinuous MLF is piecewise continuous. Based on this method, some new sufficient stability conditions were proposed for switched systems in [8]; then, the results were extended to the switched singular linear systems [9]. In [10], a type of time-varying Lyapunov function, in quadratic forms, was introduced to investigate the stability of switched linear systems. Base on this method, some sufficient conditions were derived to guarantee the globally asymptotic stability. Then, the asymptotic stability of the switched linear system in [11] with all unstable modes was studied by the method proposed in [10]. The exponentially stabilization problem for switched positive systems was investigated based on a type of multiple time-varying linear co-positive Lyapunov function method in [12].

Switched singular systems are a specific type of switched system, where each mode is represented by a singular system. Singular systems, also known as descriptor systems or algebraic differential equations, have been extensively researched by scholars. Readers can refer to citations [13,14,15,16,17,18,19,20,21] and the references therein. The singular system consists of a slowly varying dynamic part described by differential or difference equations and a rapidly varying static part described by algebraic equations [13,14]. The characteristics of singular system structure determine that it is more widely used than normal systems and has a more natural representation than normal dynamic systems [15,16]. Singular systems typically exhibit pulsing and switching behaviors characterized by abrupt changes in state or state transitions at a given time [17]. Guan et al. established necessary and sufficient conditions for the controllability and observability of a class of time-varying impulsive systems [18]. Then, the authors of [19] provided sufficient conditions for robust exponential stability in large-scale uncertain impulsive dynamic systems. The $H_{\infty }$ control problem of singular impulsive systems was discussed in [20,21]. However, the methods commonly used to study regular switched systems and singular system are generally not applicable to switched singular systems because the system state is discontinuous at the switching instant. The state jump can lead to instability or inconsistency in the system. Therefore, the study of switched singular systems should not only consider the role of switching mechanism but also consider the regularity and non-impulsiveness of singular systems. These characteristics make the study of switched singular systems a more challenging task. On switched singular systems, the stability issues for the systems with state jumps were discussed in [22]. The state jumps at the switching times were redefined using the dynamic decomposition technique in [23]. Based on the refined state jumps, new sufficient conditions for exponential stability were proposed. In [24], the theory of $H_{\infty }$ control for singular systems was extended to switched impulsive singular systems. Two controllers were designed to ensure the stability of each mode and can remove impulses when switching occurs [25]. The exponential stability and $L_{2}$ performance of discrete-time singular switched systems are considered via the multiple discontinuous Lyapunov functions [9].

However, the previous literature focused on the stability of switched systems with only stable modes or unstable modes. As far as we know, few results have been obtained regarding the stability of switched systems with both stable and unstable modes [26,27]. By introducing a unit switching sequence and sequence generator, a unified stability framework for two-dimensional discrete-time switched systems was established in [26]. The exponential stability of switched positive systems with both stable and unstable modes was discussed through a multiple piecewise continuous linear copositive Lyapunov function method in [27]. This paper focuses on the globally exponential stability of the continuous-time switched singular systems with both stable and unstable modes. Inspired by the method proposed in [10], a novel TVPLF is introduced to investigate the exponential stability under the mode-dependent average time switching rules. This method can be extended to address systems with all unstable modes.

The main contributions of this paper are as follows: (1) A novel TVPLF is proposed, which is piecewise continuously differentiable on every mode (but may not be differentiable at the interpolating points of the dwell time). This Lyapunov function method is particularly advantageous in overcoming the limitations of traditional MLF methods, which may not have a feasible solution when dealing with switched systems containing only unstable modes. (2) Dividing the mode-dependent ADT switching rules into fast and slow switching rules, by which a tighter bound of the critical dwell time is obtained. Applying the slow and fast switching rules to stable and unstable modes, respectively. (3) Based on the stability analysis, the time-varying controllers are proposed to stabilize the switched singular system, which can be expressed as the sequential linear combination of a series of linear state feedback on each mode. The proposed controllers are continuous for each mode, which are different from the controllers designed through the traditional MLF and MDLF methods, where the controllers designed by traditional MLF are time-invariant linear state feedback in each mode, while the controllers designed by the MDLF are piecewise continuous for each mode.

## 2. Preliminaries

Consider the switched linear singular system described as follows:

(1) $$E_{\sigma (t)}\dot{x}\left(t\right)=A_{\sigma (t)}x\left(t\right)$$

where $x\left(t\right)\in R^{n}$ is the state vector and $\sigma \left(t\right):R^{+}\to H=\left\{1,2,\ldots ,m\right\}$ is the switching rule, which is a piecewise constant function from the right of time and takes its values in the finite set *H*, where m≥1 is the number of the mode. $H=\bar{S}⋃\bar{U}$, where $\bar{S}$ is the set of stable modes and $\bar{U}$ is the set’s unstable modes. For a positive integer d∈H, if σ(t)=d during some time interval, it means the $d^{th}$ mode is active on this time interval. Correspondingly, the matrix $E_{d}\in R^{n\times n}$ may be singular, the rank of $E_{d}$ cannot exceed *n*, and $A_{d}$ are known real constant matrices of appropriate dimensions. For the sake of simplicity, set $\mathrm{rank}(E_{d})=r$ and $E_{d}=\left[I_{r},0;0,0\right]$, where $I_{r}$ is an *r* dimensional identical matrix and *r* is a positive integer not exceeding *n*.

**Definition** **1** ([8,9]). *During the time interval $\left[t_{0},t_{f}\right]$, denote $N_{d}\left(t_{0},t_{f}\right)$ as the number of activations for the $d^{th}$ mode, and $T_{d}\left(t_{0},t_{f}\right)$ serves the sum of the operation time of the $d^{th}$ mode. The switching rules are said to be slow switchings and have an average dwell time $T_{Ad}$ in $d^{th}$ mode if there exist two positive numbers $N_{od}$ and $T_{Ad}$ such that*

(2) $$N_{d}\left(t_{0},t_{f}\right)\leq N_{od}+\frac{T_{d}\left(t_{0},t_{f}\right)}{T_{Ad}},\forall t_{f}\geq t_{0}\geq 0.$$

*The switching rules are said to be fast switchings and have an average dwell time $T_{Ad}$ in $d^{th}$ mode if there exist two positive numbers $N_{od}$ and $T_{Ad}$ such that*

(3) $$N_{d}\left(t_{0},t_{f}\right)\geq N_{od}+\frac{T_{d}\left(t_{0},t_{f}\right)}{T_{Ad}},\forall t_{f}\geq t_{0}\geq 0.$$

**Remark** **1.** *The positive number $N_{od}$ is called the chatter bound. Inequation (2) implies the $d^{th}$ mode will be activated at most $N_{od}$ times in every time interval with the length $T_{Ad}$. Analogously, inequation (3) implies the $d^{th}$ mode will be activated at least $N_{od}$ times in every time interval with the length $T_{Ad}$. In the following sections, we adopt slow switching rules in the stable modes and fast switching rules in the unstable modes.*

**Definition** **2** ([15]). *For every d∈H, the singular system $\left(E_{d},A_{d}\right)$ is said to be*

- *regular if $det(sE_{d}-A_{d})$ is not identically zero;*
- *impulse-free if $deg\left(det\left(sE_{d}-A_{d}\right)\right)=rank\left(E_{d}\right)$.*

**Assumption** **1.** *For every d∈H, the singular system $\left(E_{d},A_{d}\right)$ is regular and impulse-free.*

This is a general assumption for singular systems.

**Definition** **3** ([9,21]). *System (1) is deemed E-exponentially stable if there exist two positive constants a,b such that the solution x(t) of the system (1) satisfies*

(4) $$\|E_{\sigma (t)}x\left(t\right)\|\leq \|E_{\sigma (t_{0})}x\left(t_{0}\right)\|\;a\exp\left(-b\left(t-t_{0}\right)\right),t>t_{0}.$$

For singular systems, E-exponential stability and exponential stability are equivalent [9,21]. With the setting $E_{d}=\left[I_{r},0;0,0\right]$, each mode is with the same dynamics decomposition form [15]. By Assumption 1, the rapidly varying static part of the state is determined by the slowly varying dynamic part. Thus, the exponential stability of the dynamic part of the system will deduce the stability of the static part. In this sense, the state jumps only affect the transient process and do not change the stability of the systems. To some extent, the state jumps can be ignored in the stability analysis with the assumptions for simplicity.

## 3. Time-Varying Piecewise Lyapunov Function

This section proposes a class of Lyapunov function, which is called TVPLF. Firstly, we divide each dwell time interval $\left[t_{i},t_{i+1}\right)$ into two subsections—that is, $\left[t_{i},t_{i+1}\right)=\left[t_{i},t_{i}+T_{ad}\right)⋃\left[t_{i}+T_{ad},t_{i+1}\right)$, where $T_{ad}$ is the critical dwell time in $d^{th}$ mode. Next, we divide $\left[t_{i},t_{i}+T_{ad}\right)$ equally into *G* segments—that is, $\left[t_{i},t_{i}+T_{ad}\right)={⋃}_{q=0}^{G-1}\left[t_{i}+J_{q},t_{i}+J_{q+1}\right)$, and every segment length is $l=\frac{T_{ad}}{G}$, where *G* is a fixed positive integer and $J_{q}=q\times l,q=0,1,\ldots ,G$. Based on the above segmentation, we construct a TVPLF:

$$V_{\sigma (t)}\left(x\left(t\right)\right)=x^{T}\left(t\right)E_{\sigma (t)}^{T}P_{\sigma (t)}\left(t\right)x\left(t\right),d\in H,$$

where $P_{\sigma (t)}\left(t\right)$ is an *n*-dimensional time-varying positive definite real matrix. For switched singular systems (1), when the switching rule switches to the $d^{th}$ mode, the above Lyapunov function is

(5) $$V_{d}\left(x\left(t\right)\right)=x^{T}\left(t\right)E_{d}^{T}P_{d}\left(t\right)x\left(t\right),d\in H,P_{d}\left(t\right)\in R^{n\times n},$$

where $P_{d}\left(t\right)$ is a time-varying matrix, which is defined as follows:

When $t\in [t_{i}+J_{q},t_{i}+J_{q+1}),q=0,1,\ldots ,G-1$

(6) $$P_{d}\left(t\right)=\left(1-\epsilon_{q}\left(t\right)\right)P_{d,q}+\epsilon_{q}\left(t\right)P_{d,q+1},$$

where $\epsilon_{q}\left(t\right)=\left(t-t_{i}-J_{q}\right)/l$ and $P_{d,q}$ are *n*-dimensional positive definite real matrices to be determined with q=0,1,…,G−1. When $t\in [t_{i}+T_{ad},t_{i+1})$,

(7) $$P_{d}\left(t\right)=P_{d,G}.$$

Owing to the above description, the TVPLF can be described as

(8) $$V_{d}\left(t\right)=\left\{\begin{array}{ccc} x^{T}\left(t\right)E_{d}^{T}\left[\left(1-\epsilon_{q}\left(t\right)\right)P_{d,q}+\epsilon_{q}\left(t\right)P_{d,q+1}\right]x\left(t\right), & \; & t\in [t_{i}+J_{q},t_{i}+J_{q+1}),\\ x^{T}\left(t\right)E_{d}^{T}P_{d,G}x\left(t\right), & \; & t\in [t_{i}+T_{ad},t_{i+1}). \end{array}\right.$$

for q=0,1,…,G−1.

**Remark** **2.** *The TVPLF has the following characteristics:*

- *The time-varying Lyapunov function depends on mode d and different modes have different functions.*
- *During the time period $\left[t_{i},t_{i}+T_{ad}\right)$, it is a linear interpolation function, whose value at the interpolation point is $x^{T}\left(t\right)E^{T}P_{d,q}x\left(t\right)$ and piecewise continuously differentiable on every mode. However, it may not be differentiable at the interpolating points of the dwell time. This is different to the general multiple Lyapunov function, which has a single constant $P_{d}$ for each mode d and is continuously differentiable during the dwell time.*

## 4. Stability Analysis

Next, we will provide the exponential stability conditions for system (1) via the TVPLF method.

**Lemma** **1** ([9]). *If $E^{T}P=PE\geq 0$, where E is a singular matrix and P is a positive definite matrix, there exists a positive matrix M such that $E^{T}P=E^{T}ME$ is satisfied.*

**Theorem** **1.** *Let system (1) satisfy Assumption 1, given the constants $\lambda_{d}<0,\mu_{d}>1,d\in \bar{S}$ and $\lambda_{d}>0,0<\mu_{d}<1,d\in \bar{U}$. If there exists $P_{d,q}>0$ in $R^{n\times n},d\in H,q=0,1,2,\ldots ,G-1$, the following conditions hold:*

(9) $$E_{d}^{T}P_{d,q}=P_{d,q}E_{d}\geq 0,$$

(10) $$A_{d}^{T}P_{d,q}+P_{d,q}A_{d}+\Theta_{d}^{q}-\lambda_{d}E_{d}^{T}P_{d,q}\leq 0,$$

(11) $$A_{d}^{T}P_{d,q+1}+P_{d,q+1}A_{d}+\Theta_{d}^{q}-\lambda_{d}E_{d}^{T}P_{d,q+1}\leq 0,$$

(12) $$A_{d}^{T}P_{d,G}+P_{d,G}A_{d}-\lambda_{d}E_{d}^{T}P_{d,G}\leq 0,$$

(13) $$E_{p}^{T}P_{p,0}-\mu_{d}E_{d}^{T}P_{d,G}\leq 0,\left(d,p\right)\in H\times H,d\neq p,$$

*where $\Theta_{d}^{q}=E_{d}^{T}\frac{\left(P_{d,q+1}-P_{d,q}\right)}{l}$. Then, system (1) is exponentially stable under the arbitrary mode-dependent ADT switching rule and satisfies*

(14) $$\left\{\begin{array}{ccc} T_{Ad} & \geq & T_{ad}=-\frac{\ln\mu_{d}}{\lambda_{d}},d\in \bar{S}\\ T_{Ad} & \leq & T_{ad}=-\frac{\ln\mu_{d}}{\lambda_{d}},d\in \bar{U}. \end{array}\right.$$

**Proof.**  For the switched singular system (1), set the $d^{th}$ mode is activated when $t\in [t_{i},t_{i+1})$ and set the candidate Lyapunov function

(15) $$V_{d}\left(x\left(t\right)\right)=x^{T}\left(t\right)E_{d}^{T}P_{d}\left(t\right)x\left(t\right).$$

On the one hand, during the dwell time period $\left[t_{i},t_{i+1}\right)$, by computing the derivative with respect to time of (15), one can derive

(16) $$\begin{array}{ccc} {\dot{V}}_{d}\left(x\left(t\right)\right) & = & {\dot{x}}^{T}\left(t\right)E_{d}^{T}P_{d}\left(t\right)x\left(t\right)+x^{T}\left(t\right)E_{d}^{T}{\dot{P}}_{d}\left(t\right)x\left(t\right)+x^{T}\left(t\right)E_{d}^{T}P_{d}\left(t\right)\dot{x}\left(t\right)\\ & = & x^{T}\left(t\right)A_{d}^{T}P_{d}\left(t\right)x\left(t\right)+x^{T}\left(t\right)E_{d}^{T}{\dot{P}}_{d}\left(t\right)x\left(t\right)+x^{T}\left(t\right)P_{d}\left(t\right)A_{d}x\left(t\right)\\ & = & x^{T}\left(t\right)\left[A_{d}^{T}P_{d}\left(t\right)+E_{d}^{T}{\dot{P}}_{d}\left(t\right)+P_{d}\left(t\right)A_{d}\right]x\left(t\right). \end{array}$$

When $t\in [t_{i}+J_{q},t_{i}+J_{q+1}),q=0,1,\ldots ,G-1$, according to (6), one can obtain

(17) $${\dot{P}}_{d}\left(t\right)=\frac{P_{d,q+1}-P_{d,q}}{l},l=\frac{T_{ad}}{G}.$$

By (6), (16) and (17), let $\Theta_{d}^{q}=E_{d}^{T}\frac{\left(P_{d,q+1}-P_{d,q}\right)}{l}$; one can obtain

(18) $$A_{d}^{T}P_{d}\left(t\right)+E_{d}^{T}{\dot{P}}_{d}\left(t\right)+P_{d}\left(t\right)A_{d}=\left(1-\epsilon_{q}\left(t\right)\right)\psi_{d,q}+\epsilon_{q}\left(t\right)\psi_{d,q+1},$$

where $\psi_{d,q}=A_{d}^{T}P_{d,q}+P_{d,q}A_{d}+\Theta_{d}^{q}$ and $\psi_{d,q+1}=A_{d}^{T}P_{d,q+1}+P_{d,q+1}A_{d}+\Theta_{d}^{q}$. Thus, by (10) and (11), when $t\in [t_{i},t_{i}+T_{ad})$, one can find that

(19) $$\begin{array}{ccc} {\dot{V}}_{d}\left(t\right)-\lambda_{d}V_{d}\left(t\right) & = & \left(1-\epsilon_{q}\left(t\right)\right)x^{T}\left(t\right)\left[\psi_{d,q}-\lambda_{d}E_{d}^{T}P_{d,q}\right]x\left(t\right)\\ & & +\epsilon_{q}\left(t\right)x^{T}\left(t\right)\left[\psi_{d,q+1}-\lambda_{d}E_{d}^{T}P_{d,q+1}\right]x\left(t\right)\\ & \leq & 0. \end{array}$$

When $t\in [t_{i}+T_{ad},t_{i+1})$, according to (12), one has

(20) $${\dot{V}}_{d}\left(t\right)-\lambda_{d}V_{d}\left(t\right)=x^{T}\left(t\right)\left[A_{d}^{T}P_{d,G}+P_{d,G}A_{d}-\lambda_{d}E_{d}^{T}P_{d,G}\right]x\left(t\right)\leq 0.$$

As a result, during the dwell time interval $[t_{i},t_{i+1}),i=0,1,\ldots$, one can derive that

(21) $${\dot{V}}_{d}\left(t\right)-\lambda_{d}V_{d}\left(t\right)\leq 0,d\in H.$$

Integrating both sides of (21) simultaneously on $\left[t_{i},t\right)$, where $t\in [t_{i},t_{i+1})$, one can obtain

(22) $$V_{d}\left(t\right)\leq V_{d}\left(t_{i}\right)\exp\left\{\lambda_{d}\left(t-t_{i}\right)\right\}.$$

On the other hand, suppose system (1) jumps from the $d^{th}$ mode to $p^{th}$ mode at the switching instant $t_{i}$. Denote $V_{d}\left(t_{i}^{-}\right)=\lim_{t\to t_{i}^{-}}V_{d}\left(t\right)$. Then, one has

(23) $$\begin{array}{ccc} V_{p}\left(t_{i}\right)-\mu_{d}V_{d}\left(t_{i}^{-}\right) & = & x^{T}\left(t_{i}\right)\left[E_{p}^{T}P_{p}\left(t_{i}\right)-\mu_{d}E_{d}^{T}P_{d}\left(t_{i}^{-}\right)\right]x\left(t_{i}\right)\\ & = & x^{T}\left(t_{i}\right)\left[E_{p}^{T}P_{p,0}-\mu_{d}E_{d}^{T}P_{d,G}\right]x\left(t_{i}\right), \end{array}$$

By observing (13), it shows that

(24) $$V_{p}\left(t_{i}\right)-\mu_{d}V_{d}\left(t_{i}^{-}\right)\leq 0$$

In the following, the Lyapunov characteristic through the entire operation process will be investigated. Assume that $t_{1},t_{2},\ldots ,t_{N}$ are switching instants of time interval $\left[t_{0},s\right]$, where *N* denotes the total number of switching times in $\left[t_{0},s\right]$. Combine (22) and (24), one can obtain

(25) $$\begin{array}{ccc} V_{\sigma (t_{N\sigma })}\left(s\right) & \leq & e^{\lambda_{\sigma (t_{N})}\left(s-t_{N}\right)}V_{\sigma (t_{N})}\left(t_{N}\right)\\ & \leq & \mu_{\sigma (t_{N}-1)}e^{\lambda_{\sigma (t_{N})}\left(s-t_{N}\right)}V_{\sigma (t_{N-1})}\left(t_{N}\right)\\ & \leq & \mu_{\sigma (t_{N}-1)}e^{\lambda_{\sigma (t_{N})}\left(s-t_{N}\right)}e^{\lambda_{\sigma (t_{N-1})}\left(t_{N}-t_{N-1}\right)}V_{\sigma (t_{N-1})}\left(t_{N-1}\right)\\ & \leq & \mu_{\sigma (t_{N}-1)}\mu_{\sigma (t_{N-2})}e^{\lambda_{\sigma (t_{N})}\left(s-t_{N}\right)}e^{\lambda_{\sigma (t_{N-1})}\left(t_{N}-t_{N-1}\right)}V_{\sigma (t_{N-2})}\left(t_{N-1}\right)\\ & \leq & \ldots \\ & \leq & \prod_{i=0}^{N-1}\mu_{\sigma (t_{i})}\exp\{\lambda_{\sigma (t_{N})}\left(s-t_{N}\right)+\lambda_{\sigma (t_{N-1})}\left(t_{N}-t_{N-1}\right)\\ & & +\ldots +\lambda_{\sigma (t_{0})}\left(t_{1}-t_{0}\right)\}V_{\sigma (t_{0})}\left(t_{0}\right)\\ & = & \prod_{d\in H}\mu_{d}^{N_{d}}\exp\left\{\sum_{d=1}^{m}\lambda_{d}T_{d}\left(t_{0},s\right)\right\}V_{\sigma (t_{0})}\left(t_{0}\right), \end{array}$$

where $N_{d}$ shows that the $d^{th}$ mode is activated $N_{d}$ times on time interval $\left[t_{0},s\right]$. $T_{d}\left(t_{0},s\right)$ describes the total running time of $d^{th}$ on time interval $\left[t_{0},s\right]$, d∈H. Because $H=\bar{S}⋃\bar{U}$, $\mu_{d}^{N_{d}}>1,d\in \bar{S}$, $0<\mu_{d}^{N_{d}}<1,d\in \bar{U}$, slow switching is used in stable modes and fast switching is used in unstable modes; combining (2) and (3), it can be found that

(26) $$\begin{array}{ccc} V_{\sigma (t_{N})}\left(s\right) & \leq & \exp\{\sum_{d\in \bar{S}}N_{d}\ln\mu_{d}+\lambda_{d}T_{d}\left(t_{0},s\right)+\sum_{d\in \bar{U}}N_{d}\ln\mu_{d}+\lambda_{d}T_{d}\left(t_{0},s\right\}V_{\sigma (t_{0})}\left(t_{0}\right)\\ & \leq & \exp\left\{\sum_{d\in H}N_{od}\ln\mu_{d}\right\}\exp\left\{\sum_{d\in H}\left(\frac{\ln\mu_{d}}{T_{Ad}}+\lambda_{d}\right)T_{d}\left(t_{0},s\right)\right\}V_{\sigma (t_{0})}\left(t_{0}\right). \end{array}$$

If $T_{Ad}$ satisfies (14), then

$$\frac{\ln(\mu_{d})}{T_{Ad}}+\lambda_{d}<0,d\in H,$$

Therefore, when s⟶∞, $V_{\sigma (t_{N})}\left(s\right)⟶0$—that is,

(27) $$V_{\sigma (t_{N})}\left(s\right)\leq \exp\left\{\sum_{d\in H}N_{od}\ln\mu_{d}\right\}\exp\left\{\sum_{d\in H}\left(\;\frac{\ln\mu_{d}}{T_{Ad}}+\lambda_{d}\right)\left(s-t_{0}\right)\right\}V_{\sigma (t_{0})}\left(t_{0}\right)$$

Because $P_{d,q}$ is positive definite, according to Lemma 1, there exist positive definite $M_{d,q}$ such that

(28) $$V_{d}\left(t\right)=x^{T}\left(t\right)E_{d}^{T}\left[\left(1-\epsilon_{q}\left(t\right)\right)M_{d,q}+\epsilon_{q}\left(t\right)M_{d,q+1}\right]E_{d}x\left(t\right).$$

It can be found that

$$\begin{array}{ccc} \underline{\lambda }{∥E_{\sigma (t_{N})}x\left(t\right)\|}_{2}^{2} & \leq & V_{\sigma (t_{N})}\left(t\right)\\ & \leq & \exp\left\{\sum_{d\in H}N_{od}\ln\mu_{d}\right\}\exp\left\{\sum_{d\in H}\left(\frac{\ln\mu_{d}}{T_{Ad}}+\lambda_{d}\right)\left(t-t_{0}\right)\right\}V_{\sigma (t_{0})}\left(t_{0}\right)\\ & \leq & \exp\left\{\sum_{d\in H}N_{od}\ln\mu_{d}\right\}\exp\left\{\sum_{d\in H}\left(\frac{\ln\mu_{d}}{T_{Ad}}+\lambda_{d}\right)\left(t-t_{0}\right)\right\}\bar{\lambda }{∥E_{\sigma (t_{0})}x\left(t_{0}\right)∥}_{2}^{2}, \end{array}$$

where $\underline{\lambda }=\min_{d\in H,q=0,1,\ldots ,G}\left\{\lambda \left(M_{d,q}\right)\right\}$, $\bar{\lambda }=\max_{d\in H,q=0,1,\ldots ,G}\left\{\lambda \left(M_{d,q}\right)\right\}$; then,

(29) $$∥E_{\sigma (t_{N})}{x\left(t\right)∥}_{2}\leq \sqrt{\frac{\bar{\lambda }}{\underline{\lambda }}\exp\left\{\sum_{d\in H}N_{od}\ln\mu_{d}\right\}}\exp\left\{\frac{\sum_{d\in H}\left(\frac{\ln\mu_{d}}{T_{Ad}}+\lambda_{d}\right)\left(t-t_{0}\right)}{2}\right\}{∥E_{\sigma (t_{0})}x\left(t_{0}\right)∥}_{2}.$$

Thus, there exist $a=\sqrt{\frac{\bar{\lambda }}{\underline{\lambda }}\exp\left\{\sum_{d\in H}N_{od}\ln\mu_{d}\right\}}>0$ and $b=\frac{-\sum_{d\in H}\left(\frac{\ln\mu_{d}}{T_{Ad}}+\lambda_{d}\right)}{2}>0$, such that

(30) $$∥E_{\sigma (t_{N})}{x\left(t\right)∥}_{2}\leq a\exp\left\{-bt\right\}{∥E_{\sigma (t_{0})}x\left(t_{0}\right)∥}_{2}.$$

Definition 3 shows that system (1) is exponentially stable. □

**Remark** **3.** *Regarding to the TVPLF, it is attenuated when the stable modes activate. However, a finite increase is allowed during the dwell time of the unstable mode. Furthermore, at the switching time, when the stable mode switches to arbitrary mode, the Lyapunov function allows a certain increase rate; when switching from unstable mode to arbitrary mode, the Lyapunov function requires a certain attenuation rate. However, from the whole operation process of the system, the decrease in the Lyapunov function can suppress the increase so as to guarantee the stability.*

**Remark** **4.** *In the process of stability analysis, we adopt the mode-dependent ADT switching strategy, utilizing a slow switching strategy for stable modes and a fast switching strategy for unstable modes. In addition, the mode-dependent ADT switching rule permits individualized ADTs for each mode rather than requiring uniform ADTs for all modes. This indicates that there are more options for the switching rules. As such, it offers greater flexibility compared with the ADT switching rule.*

**Remark** **5.** *As discussed in Remark 2, TVPLF is a linear interpolation function on every mode; thus, it is particularly advantageous in overcoming the limitations of traditional multiple Lyapunov function (MLF) methods, which may not have a feasible solution when dealing with switched systems containing only unstable modes. As such, the TVPLF offers greater flexibility in application.*

**Remark** **6.** *The parameters $\lambda_{d}$ and $\mu_{d}$ affect the feasibility of the conditions from (10)–(13). For the choice of $\lambda_{d}$, from (22), $|\lambda_{d}|$ is an upper bound of the expected divergence rate of the Lyapunov function corresponding to mode d. Thus, a larger selection of $\lambda_{d}$ will lead to possibly worse transient performance for mode d. In general, by Lemma 1, $\lambda_{d}$ can choose more than twice the largest real part of the generalized eigenvalue of mode $\left(E_{d},A_{d}\right)$. Note that $\lambda_{d}<0,\;d\in \bar{S}$ and $\lambda_{d}>0,\;d\in \bar{U}$. Roughly speaking, the larger the selection of $\lambda_{d}$, the easier it is to obtain a feasible solution to the conditions from (10)–(13). However, for a fixed $\mu_{d}$, the larger choice of $\lambda_{d}$ can result in the smaller upper bound of the MDADT of the unstable mode—that is, when $d\in \bar{U}$, the larger $\lambda_{d}$ is chosen and fewer average dwell time switching rules are available. The parameter $\mu_{d}$ describes the gap between the two Lyapunov functions before and after the switching instant. No matter whether mode d is stable or unstable, a larger selection of μ could lead to a larger feasible region for the conditions. Note that $\mu_{d}>1,\;d\in \bar{S}$ and $1>\mu_{d}>0,\;d\in \bar{U}$. However, this also will generate possibly worse transient performance for mode d and fewer choices of average dwell time switching rules for stable modes. So, there will be trade-offs of the choices of λ and $\mu_{d}$.*

## 5. Controller Design

Next, we consider the following singular switched system

(31) $$E_{\sigma (t)}\dot{x}\left(t\right)=A_{\sigma (t)}x\left(t\right)+B_{\sigma (t)}u\left(t\right),$$

where $E_{\sigma (t)},A_{\sigma (t)},x\left(t\right)$ are the same as defined in system (1), $u\left(t\right)\in R^{m}$ is the controlled input vector, and the matrix $B_{\sigma (t)}=B_{d}$ is a real constant matrix with σ(t)=d.

Considering the proposed TVPLF, a novel time-varying controller design is introduced in this section. Note that these novel controllers are sequential time-varying linear combinations of series of linear state feedback, which are continuous for each mode. They are different from the controllers designed through the traditional MLF and MDLF methods, where the controllers designed by traditional MLF are time-invariant linear state feedback in each mode, while the controllers designed by the MDLF are piecewise continuous for each mode. This is a novel contribution of this paper.

Firstly, we define the continuous time-varying linear combination state feedback $u\left(t\right)=L_{\sigma (t)}\left(t\right)x\left(t\right)$. When $t\in [t_{i},t_{i+1})$, suppose σ(t)=d,d∈H. Correspondingly, the state feedback is defined as follows:

(32) $$u\left(t\right)=L_{d}\left(t\right)x\left(t\right)=\left\{\begin{array}{ccc} K_{d}\left(t\right)x\left(t\right), & \; & t\in [t_{i}+J_{q},t_{i}+J_{q+1}),q=0,1,\ldots ,G-1\\ K_{d,G}x\left(t\right), & \; & t\in [t_{i}+J_{G},t_{i+1}), \end{array}\right.$$

where $K_{d}\left(t\right)=\left(1-\epsilon_{q}\left(t\right)\right)K_{d,q}+\epsilon_{q}\left(t\right)K_{d,q+1}$, $\epsilon_{q}\left(t\right)=\left(t-t_{i}-J_{q}\right)/l,\;q=0,1,\ldots ,G-1$. $K_{d,q},\;q=0,1,\ldots ,G$ are real constant feedback matrices to be determined.

Combining (31) with (32), we have the closed singular switched linear system

(33) $$E_{\sigma (t)}\dot{x}\left(t\right)=\left(A_{\sigma (t)}+B_{\sigma (t)}L_{\sigma (t)}\right)x\left(t\right)≜{\bar{A}}_{\sigma (t)}x\left(t\right),$$

where ${\bar{A}}_{\sigma (t)}=A_{\sigma (t)}+B_{\sigma (t)}L_{\sigma (t)}$.

Then, by Theorem 1, a similar result is obtained for the closed loop system (33).

**Theorem** **2.** *Let system (31) satisfy Assumption 1, given the constants $\lambda_{d}<0,\mu_{d}>1,d\in \bar{S}$ and $\lambda_{d}>0,0<\mu_{d}<1,d\in \bar{U}$. If there exist $K_{d,q}\in R^{m\times n}$ and positive definite $P_{d,q}\in R^{n\times n},d\in H,q=0,1,2,\ldots ,G$, the following conditions hold:*

(34) $$E_{d}^{T}P_{d,q}=P_{d,q}E_{d}\geq 0,$$

(35) $${\left(A_{d}+B_{d}K_{d,q}\right)}^{T}P_{d,q}+P_{d,q}\left(A_{d}+B_{d}K_{d,q}\right)+\Theta_{d}^{q}-\lambda_{d}E_{d}^{T}P_{d,q}\leq 0,$$

(36) $${\left(A_{d}+B_{d}K_{d,q+1}\right)}^{T}P_{d,q+1}+P_{d,q+1}\left(A_{d}+B_{d}K_{d,q+1}\right)+\Theta_{d}^{q}-\lambda_{d}E_{d}^{T}P_{d,q+1}\leq 0,$$

(37) $${\left(A_{d}+B_{d}K_{d,G}\right)}^{T}P_{d,G}+P_{d,G}\left(A_{d}+B_{d}K_{d,G}\right)-\lambda_{d}E_{d}^{T}P_{d,G}\leq 0,$$

(38) $$E_{p}^{T}P_{p,0}-\mu_{d}E_{d}^{T}P_{d,G}\leq 0,\left(d,p\right)\in H\times H,d\neq p,$$

*where $\Theta_{d}^{q}=E_{d}^{T}\frac{\left(P_{d,q+1}-P_{d,q}\right)}{l}$. Then, system (31) is exponentially stabilized by the controllers (32) under the arbitrary mode-dependent ADT switching rule and satisfies (14).*

Note that the conditions of Theorem 2 are bilinear matrix inequalities. To utilize the LMI technique, Theorem 2 can be transformed into the following version.

**Theorem** **3.** *Let system (31) satisfy Assumption 1, given the constants $\lambda_{d}<0,\mu_{d}>1,d\in \bar{S}$ and $\lambda_{d}>0,0<\mu_{d}<1,d\in \bar{U}$. If there exist $Q_{d,q}\in R^{m\times n}$ and positive definite $S_{d,q}\in R^{n\times n},d\in H,q=0,1,2,\ldots ,G$, the following conditions hold:*

(39) $$E_{d}S_{d,q}=S_{d,q}E_{d}^{T}\geq 0,$$

(40) $$\left[\begin{array}{cc} He\left(A_{d}S_{d,q}+B_{d}Q_{d,q}\right)-\left(\lambda_{d}+\frac{1}{l}\right)S_{d,q}E_{d}^{T} & \frac{1}{\sqrt{l}}S_{d,q}E_{d}^{T}\\ \frac{1}{\sqrt{l}}E_{d}S_{d,q} & -S_{d,q+1} \end{array}\right]\leq 0,$$

(41) $$\left[\begin{array}{cc} He\left(A_{d}S_{d,q+1}+B_{d}Q_{d,q+1}\right)+\left(\frac{1}{l}-\lambda_{d}\right)S_{d,q+1}E_{d}^{T} & \frac{1}{\sqrt{l}}S_{d,q+1}E_{d}^{T}\\ \frac{1}{\sqrt{l}}E_{d}S_{d,q+1} & -S_{d,q} \end{array}\right]\leq 0$$

(42) $$He\left(A_{d}S_{d,G}+B_{d}Q_{d,G}\right)-\lambda_{d}S_{d,G}E_{d}^{T}\leq 0$$

(43) $$\left[\begin{array}{cc} \mu_{d}S_{d,G}E_{d}^{T} & S_{d,G}E_{d}^{T}\\ E_{d}S_{d,G} & S_{p,0} \end{array}\right]\geq 0,\left(d,p\right)\in H\times H,d\neq p,$$

*where $He\left(A_{d}S_{d,q}+B_{d}Q_{d,q}\right)=A_{d}S_{d,q}+S_{d,q}A_{d}^{T}+B_{d}Q_{d,q}+Q_{d,q}^{T}B_{d}^{T}$. Then, system (31) is exponentially stabilized by the controllers (32) under the arbitrary mode-dependent ADT switching rule and satisfies (14) with*

(44) $$P_{d,q}=S_{d,q}^{-1},\;\;K_{d,q}=Q_{d,q}S_{d,q}^{-1}.$$

**Proof.**  (39) can be obtained by pre- and post-multiplying (34) by $P_{d,q}^{-1}$.

(42) can be obtained by pre- and post-multiplying (37) by $P_{d,G}^{-1}$.

(40) can be deduced from (35). Firstly, (35) can be rewritten as

(45) $${\left(A_{d}+B_{d}K_{d,q}\right)}^{T}P_{d,q}+P_{d,q}\left(A_{d}+B_{d}K_{d,q}\right)-\left(\frac{1}{l}+\lambda_{d}\right)E_{d}^{T}P_{d,q}+\frac{1}{l}E_{d}^{T}P_{d,q+1}\leq 0.$$

By Schur’s complement Lemma, (45) is transformed into

(46) $$\left[\begin{array}{cc} {\left(A_{d}+B_{d}K_{d,q}\right)}^{T}P_{d,q}+P_{d,q}\left(A_{d}+B_{d}K_{d,q}\right)-\left(\lambda_{d}+\frac{1}{l}\right)E_{d}^{T}P_{d,q} & \frac{1}{\sqrt{l}}E_{d}^{T}P_{d,q+1}\\ \frac{1}{\sqrt{l}}P_{d,q+1}E_{d} & -P_{d,q+1} \end{array}\right]\leq 0.$$

Then, by pre- and post-multiplying (46) by $\left[P_{d,q}^{-1},0;0,P_{d,q+1}^{-1}\right]$, one has (40) utilizing (44).

Similarly, by Schur’s complement Lemma, one can obtain (41) and (43). The proof is omitted here. □

Utilizing the LMI toolbox in Matlab, one can seek feasible controllers to exponentially stabilize system (31).

## 6. Simulation

In the following, some simulation examples are provided to verify the proposed results in Theorem 1.

**Example** **1.** *Consider singular switched linear system (1), where*

$$\begin{array}{c} E_{1}=\left[\begin{array}{cc} 1 & 0\\ 0 & 0 \end{array}\right],E_{2}=\left[\begin{array}{cc} 1 & 0\\ 0 & 0 \end{array}\right],A_{1}=\left[\begin{array}{cc} -3 & -2\\ -1 & -5 \end{array}\right],A_{2}=\left[\begin{array}{cc} -6 & -1\\ -5 & -8 \end{array}\right]. \end{array}$$

**Figure 1:**
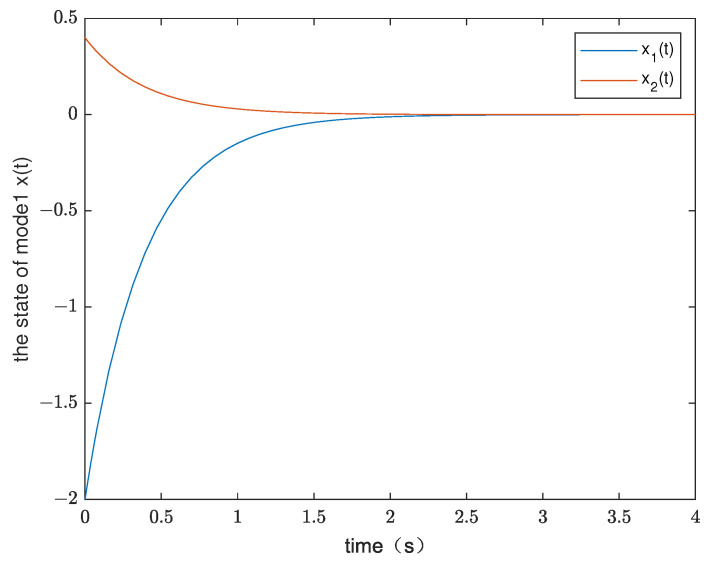
The state diagram of mode 1 in Example 1.

**Figure 2:**
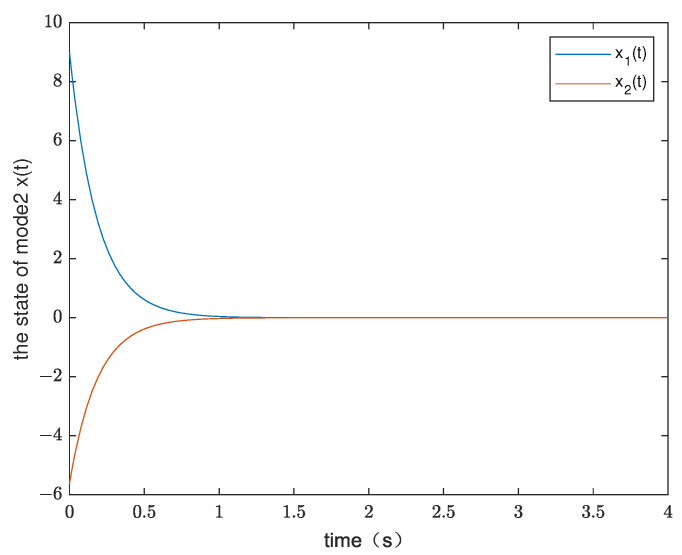
The state diagram of mode 2 in Example 1.

*By testing, the finite eigenvalues of the modes $\left(E_{1},A_{1}\right)$ and $\left(E_{2},A_{2}\right)$ are −2.6 and −5.375, respectively. Thus, the two modes of singular switched system (1) are both stable modes. The state diagram of each mode of the system is shown in Figure 1 and Figure 2. Set $G=1,\lambda_{1}=-0.8,\mu_{1}=1.02$, $\lambda_{2}=-0.9$, and $\mu_{2}=1.02$. According to (14), we obtain $T_{a1}=0.0248$ and $T_{a2}=0.0220.$ From Theorem 1, we can obtain*

$$\begin{array}{c} P_{10}=\left[\begin{array}{cc} 26.7959 & 0\\ 0 & 22.7066 \end{array}\right],P_{11}=\left[\begin{array}{cc} 30.2372 & 0\\ 0 & 18.2290 \end{array}\right],\\ P_{20}=\left[\begin{array}{cc} 21.1907 & 0\\ 0 & 11.0366 \end{array}\right],P_{21}=\left[\begin{array}{cc} 32.2894 & 0\\ 0 & 8.7422 \end{array}\right]. \end{array}$$

**Figure 3:**
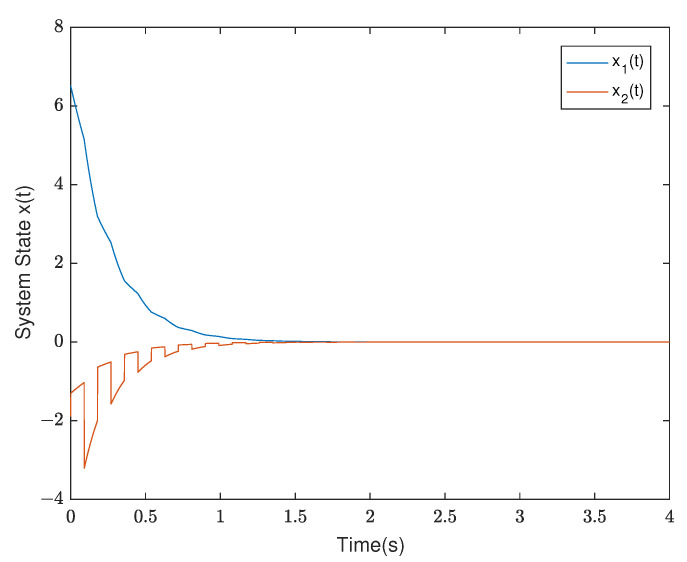
The state diagram of the system in Example 1.

**Figure 4:**
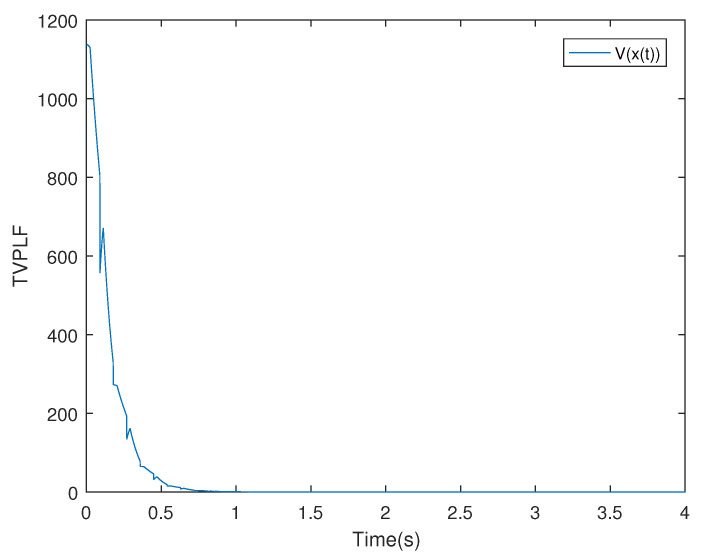
The diagram of Lyapunov function in Example 1.

*According to the switching rule (14), we choose $T_{A1}=0.03,T_{A2}=0.03$, and a compatible initial state of system $x_{0}={\left(-2,0.4\right)}^{T}$; we can obtain the system state and the trend diagram of the Lyapunov function, which are shown in Figure 3 and Figure 4. This shows that the switched singular system is exponentially stabilized by the designed mode-dependent ADT switching rule.*

**Example** **2.** *Consider the singular switched linear system (1), where*

$$\begin{array}{c} E_{1}=E_{2}=\left[\begin{array}{ccc} 1 & 0 & 0\\ 0 & 1 & 0\\ 0 & 0\; & 0 \end{array}\right],A_{1}=\;\left[\begin{array}{ccc} -1 & 0 & -1\\ 1 & -1 & 0\\ 1 & -1 & -2 \end{array}\right],A_{2}=\left[\begin{array}{ccc} -0.8415 & -1.4449 & -0.3781\\ -0.1722 & -0.4636 & 0.0122\\ -1.3446 & 0.1905 & -1.5320 \end{array}\right]. \end{array}$$

**Figure 5:**
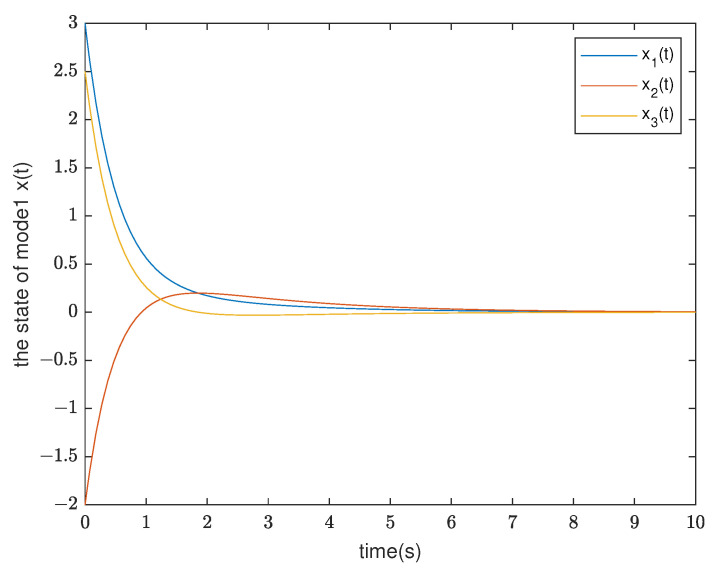
The state response of mode 1 in Example 2.

**Figure 6:**
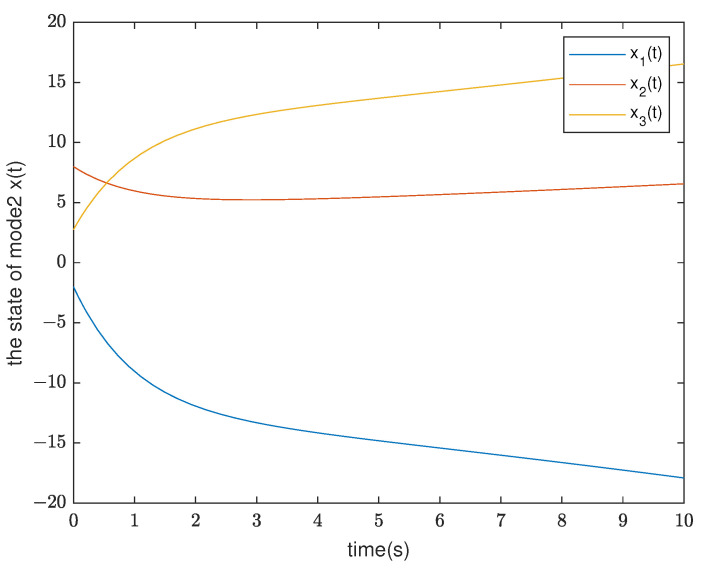
The state response of mode 2 in Example 2.

*By testing, the finite eigenvalues of the modes $\left(E_{1},A_{1}\right)$ and $\left(E_{2},A_{2}\right)$ are −2,−0.5 and −1.0088, 0.0371, respectively. Thus, the mode $\left(E_{1},A_{1}\right)$ is stable and the other is unstable. Furthermore, the state diagram of each mode of the system is shown in Figure 5 and Figure 6. Set $\lambda_{1}=-0.2,\mu_{1}=1.01$, $\lambda_{2}=0.2$, and $\mu_{2}=0.9$. According to (14), we can obtain that the minimal ADT of the stable mode is $T_{a1}=0.0498$ and the maximal ADT of the unstable mode is $T_{a2}=0.5268.$ From Theorem 1, we can choose the dwell time of $\left(E_{1},A_{1}\right)$ as $T_{A1}=0.26$ and the dwell time of $\left(E_{2},A_{2}\right)$ as $T_{A2}=0.12$. Select a compatible initial state $x\left(0\right)={\left(3.0000,-2.0000,2.5000\right)}^{T}$ and let the initial switching signal σ(0)=1. According to Theorem 1, it can be found that*

$$\begin{array}{c} P_{10}=\left[\begin{array}{ccc} 3.2849 & 0.8121 & 0\\ 0.8121 & 9.2818 & 0\\ 0 & 0 & 3.3954 \end{array}\right],P_{11}=\left[\begin{array}{ccc} 4.1690 & -0.8931 & 0\\ -0.8931 & 11.8764 & 0\\ 0 & 0 & 3.7363 \end{array}\right],\\ P_{20}=\left[\begin{array}{ccc} 3.7619 & -0.5599 & 0\\ -0.5599 & 11.3960 & 0\\ 0 & 0 & 2.5671 \end{array}\right],P_{21}=\left[\begin{array}{ccc} 3.8784 & 0.4606 & 0\\ 0.4606 & 12.4058 & 0\\ 0 & 0 & 0.9887 \end{array}\right]. \end{array}$$

**Figure 7:**
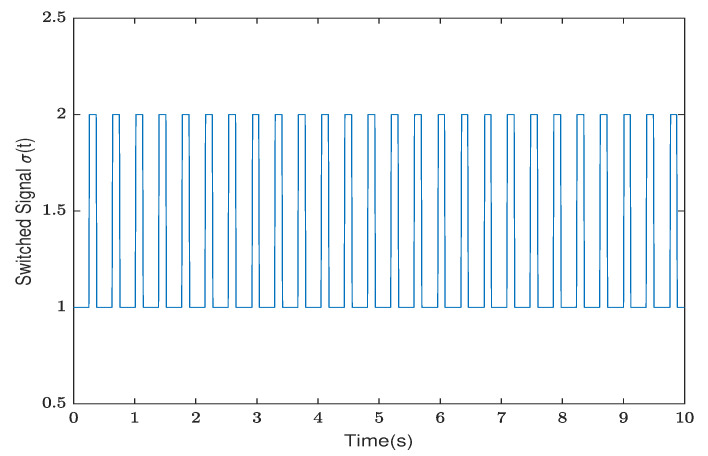
The switching rule of the system in Example 2.

**Figure 8:**
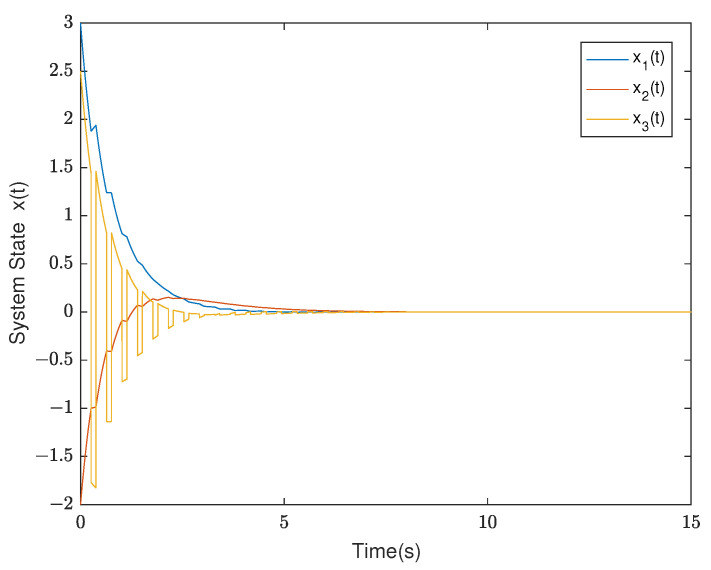
The state diagram of the system in Example 2.

**Figure 9:**
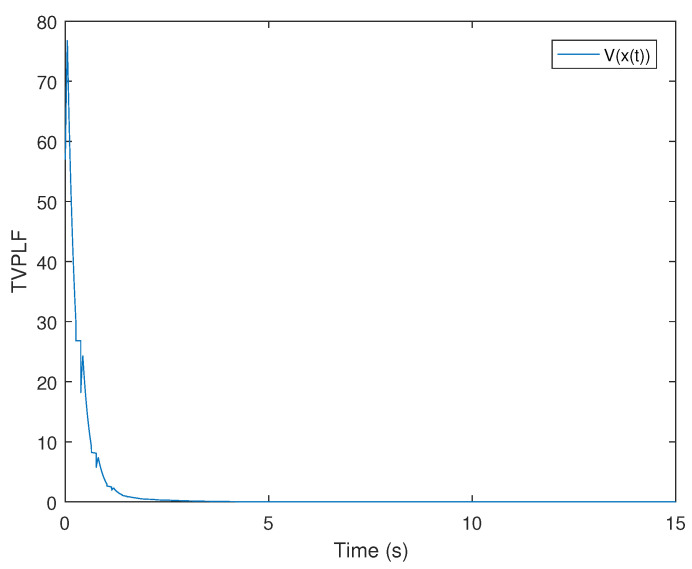
The trend diagram of TVPLF in Example 2.

*The diagrams of the switching rule of the system and the state response under the switching rule are shown in Figure 7 and Figure 8, respectively. It can be observed that the system is stabilized after about 7 s under the mode-dependent ADT switching rule, which verifies its effectiveness. The trend diagram depicting the TVPLF is presented in the right subfigure of Figure 9.*

**Example** **3.** *Consider the singular switched linear system (1), where*

$$\begin{array}{c} E_{1}=\left[\begin{array}{ccc} 1 & 0 & 0\\ 0 & 1 & 0\\ 0 & 0\; & 0 \end{array}\right],A_{1}=\left[\begin{array}{ccc} -0.8415 & -1.4449 & -0.3781\\ -0.1722 & -0.4636 & 0.0122\\ -1.3446 & 0.1905 & -1.5320 \end{array}\right],\\ E_{2}=\left[\begin{array}{ccc} 1 & 0 & 0\\ 0 & 1 & 0\\ 0 & 0\; & 0 \end{array}\right],A_{2}=\left[\begin{array}{ccc} -1.2435 & 0.8775 & -0.9747\\ -0.7409 & -1.5072 & -0.8519\\ -1.5536 & -0.6242 & -1.0759 \end{array}\right]. \end{array}$$

*It is easy to test whether the finite eigenvalue of mode $\left(E_{1},A_{1}\right)$ and $\left(E_{2},A_{2}\right)$ are −1.0088,0.0371 and 0.0155,−0.8645, respectively. So, these two modes are all unstable. According to the discussion in the introduction, there is no feasible MLF for this switched singular system. However, by choosing $\lambda_{1}=0.3,\mu_{1}=0.85$, $\lambda_{2}=0.2$, and $\mu_{2}=0.9$, according to (14), we can obtain that the maximal ADTs of these two modes are $T_{a1}=0.5417$ and $T_{a2}=0.5268$, respectively. From Theorem 1, we can choose the dwell time of $\left(E_{1},A_{1}\right)$ as $T_{A1}=0.4417$ and the dwell time of $\left(E_{2},A_{2}\right)$ as $T_{A2}=0.4268$. Select a compatible initial state $x\left(0\right)={\left(3.0000,-2.0000,-2.8817\right)}^{T}$ and let the initial switching signal σ(0)=1 and G=1. According to Theorem 1, there exist feasible $P_{d,q},d=1,2,q=0,1$ satisfying conditions from (9)–(13) as follows:*

$$\begin{array}{c} P_{10}=\left[\begin{array}{ccc} 0.9180 & -0.8463 & 0\\ -0.8463 & 2.7404 & 0\\ 0 & 0 & 0.7071 \end{array}\right],P_{11}=\left[\begin{array}{ccc} 1.1653 & -0.5058 & 0\\ -0.5058 & 2.8514 & 0\\ 0 & 0 & 0.4884 \end{array}\right],\\ P_{20}=\left[\begin{array}{ccc} 0.9678 & -0.4943 & 0\\ -0.4943 & 1.9892 & 0\\ 0 & 0 & 0.5707 \end{array}\right],P_{21}=\left[\begin{array}{ccc} 1.0256 & -0.9348 & 0\\ -0.9348 & 3.2343 & 0\\ 0 & 0 & 0.5234 \end{array}\right]. \end{array}$$

**Figure 10:**
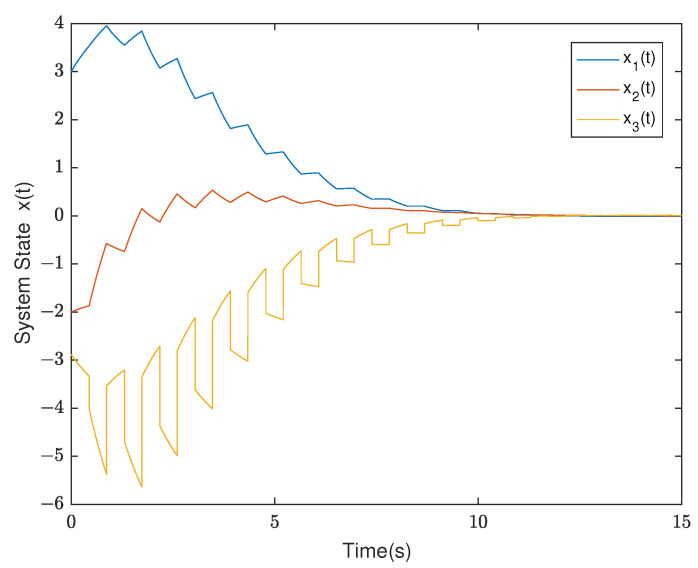
The state diagram of the system in Example 3.

**Figure 11:**
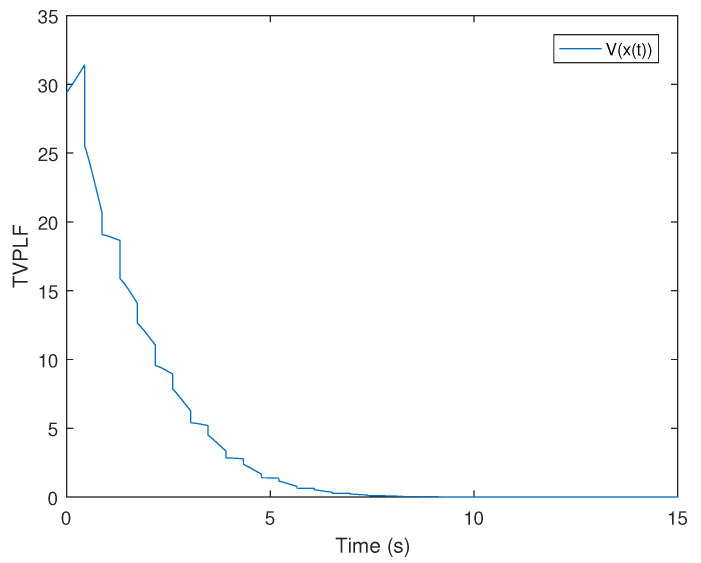
The trend diagram of TVPLF in Example 3.

*Thus, compared with the traditional MLF approach, the TVPLF method is less conservative. Figure 10 and Figure 11 show the state response and the TVPLF evolution.*

## 7. Conclusions

This paper analyzes the exponential stability of a type of switched singular linear system through a novel TVPLF method, and some computable sufficient conditions are obtained for the exponential stability under mode-dependent ADT switchings. Based on the stability analysis, a series of novel time-varying controllers are designed to stabilize the switched singular systems. These controllers are continuous and can be expressed as a sequential linear combination of a series of linear state feedback on each mode. This method can be extended to general switched systems for stability analysis, gain performance analysis and control design, and so on.

## Data Availability

Not applicable.
